# The Hospital Saturday Fund's System of Distribution

**Published:** 1897-04-17

**Authors:** 


### Editor's Letter-Box.

[Our Correspondents are reminded that prolixity is a great bar to publication, and that brevity of style and conciseness of statement greatly facilitate early insertion.]

### THE HOSPITAL SATURDAY FUND'S SYSTEM OF DISTRIBUTION.

Mr. R. B. D. Acland writes: I think if you look at the resolution which I moved at the Mansion House you will see your memory is at fault. My motion was for a committee to inquire whether any alteration should be made in the clause to which you refer, and if my memory serves me well I did not once mention the Saturday Fund system of distribution in the course of my speech, though I criticised the Sunday Fund system to the best of my ability. However, this is a small matter, and as you correctly represent my position when you say that I want the Hospital Saturday Fund to be a help to the hospitals and a credit to everybody concerned, I should be content to let the matter rest were it not for the suggestion contained at the end of your editorial note on my communication that I should resign the chairmanship of the Fund because the annual street collection is to be held this year. That is a suggestion I cannot adopt, just because my position is as you represent it. I believe the Hospital Saturday Fund has done in the past and will do in the future most useful work, though I am fully conscious that as a body it has made many mistakes, to some of which I have been a party. But I do not conceive it to be the duty of a chairman to resign his office in an organisation which he believes capable of useful work simply because that organisation takes a course which does not commend itself to him, unless that course amounts to something absolutely wrong. To do so seems to me would amount to that weakness which you think I am perilously near, or rather I should prefer to say to downright cowardice. These questions of street collections and methods of distribution are just cases in point. A street collection in essentials is nothing but meritorious, for no one in his senses can say that for a number of ladies to band themselves together to collect alms from passers by for a great system of charity from which tens of thousands yearly receive the greatest benefits, is one whit more immoral than gathering contributions by appeals sent by the post or advocated with all the prestige which attaches to great names by every newspaper in the land. But they become undesirable under certain conditions, and in your opinion and mine those conditions now exist and cannot be removed. The duty of a chairman under these circumstances seems to me patiently to work away until he can secure the abolition of that which has become undesirable, because it is *undesirable*, not because it is bad in itself. This is the way in which every great reform has been achieved in the past and will be achieved in the future, and not by the school boy method of "Very well, if you won't do what I want I shan't play any more." So with regard to the distribution. Our system is far from perfect. We know it, and are working hard to improve it. At present we know of none better, for the Sunday Fund system, the only one which attempts to deal with the problem presented to the Saturday Fund, produces results nearly as "curious." Compare, for instance, the grant of £889 made in 1895 to Guy's Hospital with that of £2,062 made the same year to St. George's. There being, therefore, no system ready to our hand, we are trying to work one out which shall avoid defects we are aware of and yet continue what is good in our old system. Here again I believe that a man who has it in his heart to do the best he can is a better citizen if he tries to improve rather than if, finding the matter difficult, he gives up the attempt.

The difficulty of effecting reforms in the two matters to which you refer is very great, but that does not make the attempt any the less attractive. These difficulties would not be increased if our critics were to attempt to appreciate the disadvantages under which the Hospital Saturday Fund has laboured. Founded by men unaccustomed to hospital management, it has succeeded in attracting the support of a very large number of persons, and has of recent years steadily increased. A system of distribution has been established in the main, I believe, on right lines, which fails, as we are well aware, in certain matters. That it fails in so few is a matter of marvel to me when I remember that from first to last the delegates have received practically no assistance. But we are learning our lesson as all have to do, and I hope that one result of our present reconsideration of our system of distributing our funds will be that you and our other critics will admit that we have made a serious contribution to the difficult question of how most fairly to distribute a large sum of money among more than 150 institutions. Meanwhile, I confess that I think that while this serious attempt is being made, the "monstrous," "educated man," "improper application of funds" style of criticism is rather out of place. Only one word more. I agree that at last a time comes when attempts to improve matters must be given up if one has any regard for his self-respect; but, in my opinion, that time has not yet nearly come for me.

